# GP referral to rapid diagnostic centres for non-specific cancer symptoms: a qualitative study

**DOI:** 10.3399/BJGPO.2024.0179

**Published:** 2025-08-13

**Authors:** Caroline White, Spencer Robinson, Una Macleod, Charlotte Kelly

**Affiliations:** 1 Hull York Medical School, University of Hull, Hull, UK; 2 Humber and North Yorkshire Cancer Alliance, Hull, UK; 3 Hull York Medical School, University of Hull, Hull, UK

**Keywords:** cancer, neoplasms, GP referral, rapid diagnostic centre, general practitioners, qualitative research

## Abstract

**Background:**

Diagnosing cancer can be challenging, especially when patients present to GPs with serious, but non-specific symptoms. Rapid diagnostic centres (RDCs) have been introduced in England as diagnostic pathways for patients with non-specific symptoms where cancer is suspected, but they do not meet existing cancer pathway criteria.

**Aim:**

To investigate GP perspectives on referral to an RDC pathway for patients with non-specific symptoms and suspected cancer.

**Design & setting:**

A qualitative study using semi-structured interviews with GPs, within the catchment area of an acute NHS trust in the UK.

**Method:**

GP interviews focusing on experiences of using the RDC pathway. A thematic analysis was conducted on interview transcripts.

**Results:**

GPs reported the RDC pathway as a game changer. It offered faster referral, reduced anxiety for GPs and patients, and reduced the need for GPs to ‘*game the system*’ when patients do not meet criteria for cancer-specific pathways. The narrative required on referral appeared to legitimise GP gut feelings and expertise. RDC results (if not cancer) gave GPs space to treat patients without concern of a missed cancer, while ensuring onward referral for those with cancer or other serious conditions. Some access barriers, especially related to travel and time, were identified especially for patients in rural areas.

**Conclusion:**

This cancer pathway fills a referral gap for GPs and patients with non-specific potential cancer symptoms. It has an important signposting function, helping identify patients requiring treatment for cancer or other serious conditions, and others whose symptoms can be treated safely within primary care.

## How this fits in

Previous research has explored the rapid diagnostic centre (RDC) pathway from a secondary care setting, indicating the benefit to diagnosing cancers and other non-cancer diagnoses. This study aimed to explore the pathway from the perspective of the GPs referring into the RDC pathway and how it supports decision making in primary care. It was seen by some GPs as a game changer providing them with quicker access to testing, diagnosis, and treatment for patients whose symptoms were concerning, but not specific, and reducing anxiety both to GPs and patients. This study highlights the anxiety for patients and GPs about potential cancers; this new diagnostic pathway helped GPs determine when patients could be safety treated (without concern about a missed cancer) in primary care, and when onward referral for further investigation and/or treatment was warranted.

## Introduction

The journey to cancer diagnosis is often complex, with GP cancer symptom recognition underpinned by uncertainty.^1,2^ While relatively few GP consultations result in cancer diagnosis, considerations of possible cancer are the daily experience in primary care, occupying considerable time and causing concern about potential missed diagnoses.^1^ There are particular diagnostic complexities for patients with non-specific symptoms, such as unexplained weight loss, fatigue, non-specific pain, loss of appetite, which may be symptoms of cancer or of multiple other conditions, as well as signs of ageing or frailty.^2–7^ Determining the significance of these symptoms is challenging, and has been hampered by a lack of appropriate pathways for timely investigation, while in the UK there is ongoing concern about rates of cancer mortality and levels of diagnosis of cancer at stages 1 and 2 have stalled.^8^ Thus, patients with non-specific symptoms are at risk of multiple consultations, delayed or late diagnoses, and poorer outcomes.^1,2,9^

Following developments in Denmark,^10^ rapid diagnostic centres (RDCs) have been established in the UK, Sweden, and Norway to support urgent referral and diagnosis on the basis of non-specific symptoms or GP gut feeling.^2–4,11,12^ Early research indicates that RDCs facilitate diagnosis of cancers at all stages, including rare cancers, which can present particular diagnostic challenges, and of multiple non-cancer conditions.^2–4,11^ However, to date, there has been limited research from the perspective of GPs, who are the main referrers to RDCs.^11^ This study was one of the first to provide insights into GPs’ use of an RDC pathway and explore how access to this shapes their referral behaviours and decision making. Since NHS England^13^ is committed to ensuring RDCs have 100% population coverage, it is particularly important to explore the extent to which GPs engage with and refer to RDCs, and any barriers to referrals and patient attendance. GPs as the referrers into RDCs are essential to their success.

### The RDC pathway

This was a qualitative study of an RDC launched in 2020 by an NHS trust, covering a geographic footprint that included a small city, coastal towns and villages, and a large rural population. There is variation among the RDC diagnostic pathways for non-specific symptoms across different NHS trusts.^3,11^ Within the geographical area studied, access to the RDC is via GP referral, following preliminary testing in primary care. GPs in this geographical area have access to the ‘ICE button’, a single point of reference that enables them to order a suite of predetermined tests online (for example, bloods, faecal immunochemical test [FIT]). The ICE button has been developed as part of the RDC pathway in this setting and is not routinely included as a standard part of the RDC pathway in all NHS trusts. Referral criteria are summarised in Table 1.

**Table 1.** Criteria for rapid diagnostic centre referral

Referral criteria
Patient aged 40 years or over
New, unexplained, unintentional weight loss
Unexplained constitutional symptoms, including loss of appetite, fatigue, nausea, malaise, bloating
Unexplained abdominal pain
Unexplained or progressive pain
GP gut feeling of potential cancer

On the basis of test results patients may be referred to the RDC, with test results and a narrative of GPs’ concerns required. Following review of the referral to ensure all tests have been conducted within the required timescales (4 weeks for blood tests, 8 weeks for FIT), patients go ‘straight to test’, usually computed tomography (CT) scans (of chest, abdomen and pelvis), with other tests determined by the consultant. Test results are reviewed at multidisciplinary RDC team meetings, and patients then attend the RDC clinics at one of two district hospitals and meet with a consultant or nurse specialist. Diagnoses (cancer or non-cancer) may be given at this appointment and onward referrals made where relevant.

## Method

### Participant recruitment

GPs working within the geographic footprint of the NHS trust were eligible to participate if they had used the RDC ICE button at least once. However, two GPs who had not used the ICE button also participated, providing insights into some of the reasons for non-use. GPs received emails about the study from the RDC lead and GP cancer leads, the study was publicised in primary care newsletters, and the researchers contacted all practice managers within the RDC footprint with study information.

### Data collection

GPs were interviewed online, by phone or in-person, depending on personal preference, using a semi-structured interview schedule. This included questions about their actions when patients have potential cancer symptoms, especially when signalled by non-specific symptoms; their experiences of using the ICE button and referral to the RDC; and the circumstances in which they use the pathway. GPs were also asked about any factors they perceive influence patient attendance at RDCs. Interviews were transcribed and anonymised. Participants were given a continuing professional development certificate and a £50 shopping or charity voucher to acknowledge their participation.

### Data analysis

Interview transcripts were analysed thematically, following the analytic stages outlined by Braun and Clarke,^14^ to identify key themes underpinning GP referral decisions and behaviour. Following familiarisation with the data through detailed reading of transcripts, a sample was coded independently by two researchers. Codes were drawn inductively from the data, enabling identification of provisional themes, which were refined through further team discussion. All transcripts were coded using NVivo (version 14).

## Results

Thirteen GPs were interviewed between September 2023 and January 2024 of whom 62% were female. Twenty-three per cent were GP partners; 54% salaried GPs and 8% locums (14% missing data). Thirty-one per cent had qualified in 2020 or later, 54% between 2000 and 2019, and 15% before 2000. Forty-six per cent practised in city practices (where the main RDC clinic was conducted), 39% in rural locations and 15% in coastal towns.

Themes identified related to the following: (1) cancer as an ever-present possibility, yet hard to diagnose*;* (2) the journey to diagnosis: the role of the RDC pathway in supporting GP decision making when cancer is suspected*;* (3) the reassurance of an alternative pathway: the role of the RDC in supporting GPs and patients; (4) GP knowledge and awareness of the RDC pathway; and (5) GPs perceive positive impacts for patients, but some barriers to attendance were highlighted.

### Cancer as an ever-present possibility, yet hard to diagnose

The first theme set the context for the study from the perspective of our GP responders. It highlights that the possibility of cancer appeared to occupy GPs’ thoughts and they were mindful of the prospect of missed or late diagnoses:

*‘With every consultation I try and have just that little mental check list of the "what ifs". What if it could be cancer, what do I have to ask about that might indicate a serious disease here?’* (GP13)

GPs experienced uncertainty in determining when symptoms might be owing to cancer, with patient symptoms, primary care practice, and patient-related factors all challenging diagnosis. Diagnostic challenges were particularly acute when patients had non-specific symptoms, which could be attributable to multiple causes (including other physical and mental health conditions, ageing, and frailty), and sometimes concerns could be signalled by ‘gut feelings*‘*:

*‘Sometimes there’s not much else to go on other than this person just looks like they’re really failing and we don’t really know why.’* (GP11)

In such circumstances it was unclear whether symptoms were indicative of cancer, which organs were affected, and which referral pathways should be followed, while being aware that ’*you don’t want to refer everyone’* (GP01)*,* risking overwhelming cancer diagnostic pathways.

Challenges in respect of current primary care practice were identified. Patient continuity was considered to facilitate observation of changes and concerning signs, and enabling patients to feel comfortable raising concerns. However, a loss of continuity was reported in some practices:

*‘If you’ve got the continuity, you can go — I know what’s going on, I know you, or I know your family history … I know what’s normal for them. But that’s increasingly gone out of the window. And that is a major issue for picking up cancer where there’s not obvious red flags.’* (GP08)

Recent changes in primary care, including increasing remote consultations, reliance on other professionals, such as advanced care practitioners, to conduct some examinations, as well as insufficient time to take and review complex histories, were all felt to further confound cancer diagnosis. Patients were reported to attend with multiple symptoms and delayed presentations, attributed to increasing challenges getting appointments, patient stoicism, and not wanting to bother the GP. This resulted in GPs having to interpret multiple symptoms and increased risks of late diagnosis.

### The journey to diagnosis: the role of the RDC pathway in supporting GP decision making when cancer is suspected

Eleven participants had used the ICE button at least once. They reported that it facilitated the process of ordering tests and was a useful *aide memoire,* reminding them of the required tests, and prompting them to think more widely about patient symptoms.

GPs used the ICE button in a range of circumstances including the following: in response to concerning patient symptoms, with weight loss frequently reported; a troubling patient or family history, especially a history of cancer or smoking; when they had a strong suspicion of cancer, but the site was unclear; when they could not find an explanation for symptoms or identify the source; and, on occasion, in response to patient anxiety and to reassure.

There was some frustration about the requirement for recent test results, which could necessitate repeat testing:

*‘It can sometimes take a little bit of time to work them up and decide what you’re going to do … you could start by doing some blood tests and then you could maybe see them a couple of weeks later … And it could be a couple more weeks before you decide to do that referral at which point the tests have expired … so you have to then redo all of the tests, which sets you back further.’* (GP02)

This might be a disincentive to watch and wait, or put a limit on how long GPs are prepared to do so, to ensure the initial tests can be used.

Test results influenced GP decisions. Onward referral to the RDC was undertaken when patients had negative test results coupled with ongoing symptoms, GP concern, and/or a cancer history, or when test results did not meet thresholds for other pathways:

*‘All the investigations were okay, but the patient was losing weight drastically …. there wasn’t anything to suggest the cause … after having done the routine investigation in the practice, because I wouldn’t know whether to go through the respiratory pathway or the colorectal pathway … plus there was no other history apart from the weight loss and their worry about that. So I did the RDC.’* (GP10)

However, negative results, coupled with relatively low-level concerns, could reassure GPs that further referral was not required, or that they could watch and wait:

*‘If it’s a patient where there was a suspicion of something, but not really a massive concern … they are reassured, and they feel better. And we sometimes say, look the investigations have actually not shown anything, but we know that there’s a lot of things it’s not.’* (GP03)

Positive test results could indicate alternative referral routes were required, diverting patients from the RDC pathway, highlighting the role of the tests in signposting to optimal pathways:

*‘The FIT test result came back and it was positive which we weren’t necessarily expecting … We ended up using the lower GI Two Week Wait Pathway instead. So even though we’d done the full RDC ICE proforma we ended up changing tack with the results.’* (GP12)

In other instances, positive results indicated problems that could be managed within primary care, with watch and wait or no further referral indicated. GPs also reported not referring for further investigations if symptoms resolved, or it was considered that patients would not benefit from further investigation, for example, if very frail.

The process of referral was reportedly *easy* and ’*straightforward’*. Unlike other 2-week wait (2WW) pathways, a GP narrative was required. One GP noted that this made referral more onerous, especially if required to collate information from consultations with multiple GPs. However, others appreciated the opportunity to articulate their concerns and appeared to feel this lent legitimacy to their insights:

*‘Sometimes it is just a gut feeling, you know something’s not right but you can’t put your finger on it … There is a free text bit so that you can actually write exactly … what the story is and what your concerns are … It gives you an opportunity to give some context as well as the objective* [test results]*.’* (GP12)

Participants were asked about the outcomes of RDC investigations, although not all were known at the time of interview. Cancer diagnoses were seldom mentioned: instead other diagnoses were reported, including serious and hard-to-diagnose conditions. This was found helpful, and non-cancer diagnoses were reportedly a relief to patients and GPs alike, giving space to focus on treatment or alternative management strategies without a background anxiety about potential cancer or missed diagnosis:

*‘The answer could be* [they’ve] *lost* [significant] *weight and actually it’s because* [they are] *really struggling with anxiety at the moment and that’s the main issue. So the clinic isn’t going to fix that … but it’s going to allow me as a clinician to then say "phew I’m not dealing with cancer or anything serious, I can now do some serious work with* [their] *mental health and try and get … some help on that domain".’* (GP06)

### The reassurance of an alternative pathway: the role of the RDC in supporting GPs and patients

The RDC provided a welcome referral pathway when GPs were concerned about patient symptoms, but they did not meet the criteria for other pathways or it was not clear which was most appropriate:

*‘Sometimes we’re stuck in this middle ground of — well they don’t fit this box and they don’t fit this one and they don’t fit that one. But I’m stuck now and I have nowhere to go with them.’* (GP11)

In common with other areas,^15,16^ the colorectal 2WW pathway had been amended requiring a positive FIT. GP views about this changed requirement varied, with mixed views about the robustness of the evidence base and risks of missed cancers. However, the RDC gave GPs an alternative referral pathway when results were FIT negative, but they were concerned about possible cancer. The continued access to a rapid referral pathway afforded by the RDC may have helped allay GP misgivings and increased the acceptability of this change for some.

GPs considered the RDC provided faster access to testing, which could expedite diagnosis and treatment, as well as access to expert medical opinion and full assessment of patient symptoms. The relatively open referral criteria could also reduce the need to ‘game the system*‘* so that patients about whom GPs are concerned are seen within cancer-specific pathways:

*‘I wish I’d had it for* [previous patient] *… I spent hours agonising … I knew they needed a two-week wait … I really fudged the numbers to get them into that* [pathway]*. And the RDC would have been perfect.’* (GP05)

The pathway afforded reassurance to GPs; for example, a newly qualified GP conceptualised the RDC as a ’*refuge’,* offering support in the context of potentially overwhelming diagnostic uncertainty:

*‘It’s very wonderful to know that you have at your back as a clinician working with doubt … you actually come across a lot of uncertainty. So sometimes those uncertainties can be way too much … and gut instinct is saying something is going wrong. So I think I see RDC as a refuge.’* (GP10)

While GPs primarily reported positive experiences, some also highlighted concerns and risks. Some expressed concerns that the availability of the pathway might stifle GPs’ diagnostic skills and confidence, providing them with ’*an easy get out place for handing over that responsibility to somebody else’* (GP05)*,* perceiving it as ’*lazy’* and a ’*tick-box exercise’* (GP09). This was a disincentive to use the pathway for one GP. Risks of over-referral, overwhelming the clinic, and disadvantaging those about whom there is greatest concern were also identified.

### GP knowledge and awareness of the RDC pathway

While most participants had received information about the RDC, others did not appear to have received timely information. One was unaware of the RDC and had consequently never used the ICE button or referred to the RDC, although they considered ’*it would be transformative’* (GP08). Another had used the pathway but had not initially known about the ICE button:

*‘I actually don’t think I realised it was there for quite a while of doing the referrals, I was just doing the panel of bloods from my own head.’* (GP01)

Some noted they were not aware of what happened to patients at the clinics, apart from a general awareness they were likely to have scans, and there were apparent difficulties for some in keeping abreast of pathway changes.

### GPs perceive positive impacts for patients, but some barriers to attendance were highlighted

Participants were confident that patients would attend the RDC, perceiving patients presenting with non-specific symptoms were concerned about their health and symptoms, and motivated to undergo further testing:

*‘Usually you’re not fighting for these people to go get seen. They are people who want answers and are worried about their vague symptoms, and they are willing to take more steps to get this sorted.’* (GP03)

However, travel was identified as a barrier for some, and could be especially acute in some rural areas. While some patients were felt to accept this as an aspect of rural life they were accustomed to managing, for others distance presented challenges associated with the costs and time required to travel, lack of access to own transport, coupled with limited public transport. Travel therefore represented:

*‘One of the biggest issues, even to the point where it makes the decision for some people as to whether they actually want to be referred … that’s often something they worry about more than the referral itself sometimes.’* (GP12)

Time burdens were associated with travel, time spent at clinic, and at future appointments for further tests or treatment if required. A recent relocation of scan appointments from the city to an outlying town was felt to present difficulties both for rural patients, who would have more difficult journeys, and city-based patients, who hitherto appeared to have experienced fewer travel barriers than their rural and coastal counterparts.

The research did not encompass patients’ perspectives. However, GPs perceived that patients welcomed the RDC referral, appreciating their concerns being taken seriously and actions being undertaken to investigate and resolve their concerns. As patients were often felt to be anxious about their symptoms, they appeared to value the speed at which appointments were arranged, which was perceived as providing a relatively short timeframe for managing anxiety; and non-cancer diagnoses could provide relief and reassurance.

*‘I rang him up and …“Oh, I’ve been told there’s no cancer and that.” So he was just happy … just happy to know that … there’s probably a hundred, thousands of other words said but the bit that really mattered to him was that.’* (GP11)

## Discussion

### Summary

There are inherent challenges in determining when non-specific symptoms might signal cancer, and previously GPs have lacked access to timely referral options when they are concerned about patients with such symptoms. In this context, the RDC pathway provided a welcome and rapid approach to investigating non-specific symptoms of concern, and access to a referral route when patients were ineligible for other pathways. The initial tests helped GPs identify whether onward referral for further investigation was warranted, and when it was appropriate to treat locally or watch and wait. Where FIT results were negative, the RDC provided an alternative to the 2WW colorectal pathway for patients with concerning symptoms; the availability of the RDC may have helped allay the concerns of some GPs about the changed requirements for the colorectal pathway. The RDC supported diagnosis of cancer, other, often hard-to-diagnose conditions, as well as those which GPs could treat locally without a backdrop of anxiety about an underlying cancer. Thus, the RDC has an important signposting function, ensuring that patients are correctly situated on a cancer pathway or are treated elsewhere (where necessary). Although the pathway had been widely publicised, not all appeared aware of this, highlighting the need for ongoing efforts to maintain GP awareness and referrals. GPs perceived that patients welcomed referrals and their symptoms being taken seriously, and were motivated to attend. However, barriers associated with time to travel and attend clinics were identified.

### Strengths and limitations

To date there has been limited attention within RDC-focused research on GP perspectives, although Vasilakis and Forte^2^ did include GP views. They have important roles in patient referral, meaning that pathways need to be acceptable and accessible to them. The current study conducted in-depth interviews with GPs, exploring their interactions with the pathway, how it shapes their decision making, and their experiences of using it. Fewer GPs than envisaged participated, likely reflecting the intensity of pressures and workload in primary practice. Among participants, city-based GPs, whose patients were in close proximity to the main RDC clinic, were well represented. However, relatively few GPs from coastal practices participated; some of the areas of highest deprivation within the health trust area are coastal, meaning important perspectives, including those that may impact on attendance, are not reflected. As there is diversity among RDC pathways, the findings may not be applicable to other areas, highlighting a need for further research with GPs elsewhere.

### Comparison with existing literature

GPs confirmed the difficulties reported elsewhere in determining whether non-specific symptoms may signify cancer. In addition to symptom-related challenges, participants highlighted diagnostic challenges related to contemporary primary care practice. These included the growing lack of patient continuity; continuity is valued by clinicians and patients alike, and facilitates GP observations of change and significant illness.^6,17,18^ Increased use of remote consultations were felt to be a further barrier to observing subtle signs that may signal the need for further investigation.^19,20^ Our findings reflect the challenges inherent in modern-day general practice: of providing whole patient-centred care in the context of pressures to reach diagnosis and to manage the complexities of systems that impact on primary care, in a context of heavy workloads.^21^ Difficulties making appointments and concerns about troubling the doctor, both previously identified,^22,23^ were felt to contribute to late presentation of symptoms, and of multiple symptoms. Green *et al* ^1^ concluded that cancer occupies a disproportionate amount of GP time and resources; our findings suggest that this includes GP emotions and ‘*head-space*’, with the possibility of cancer seemingly an almost ever-present thought. Alongside the pressure not to miss cancer, GPs also appear anxious not to over-refer and overwhelm pathways,^24,25^ highlighting the finely-tuned decisions required. In this complex and challenging practice context, the RDC appeared to provide a valued approach to addressing diagnostic uncertainty.

The RDC pathway was viewed positively by almost all GPs interviewed. Testing and referral were experienced as straightforward, and they valued having an alternative referral pathway for patients with non-specific symptoms, reducing the need to ‘*game the system*’ to get patients seen when concerned. Their views align with the ’*overwhelming satisfaction’* reported by GPs in Vasilakis and Forte’s^2^ study, who also valued the ease of referral, rapidity of the pathway, and experienced reduced stress owing to having an additional referral option. GP gut feelings play an important role in highlighting when patients may require further investigations^1,24^ and the RDC pathway and the inclusion of the GP narrative on referral appeared to validate GPs’ gut feelings and judgements that patients should be investigated.

The RDC serves a large rural population, often located at a distance from the clinics. Distance and travel have been previously explored and findings are equivocal; however, there is some evidence that distance and travel may be significant for some.^26–28^ The findings suggest that there may be a complex interplay between distance to clinics; ability to drive or access public transport; time to travel and time required at clinics; poverty or affluence; and age and frailty. Previous research identified further barriers to attendance for urgent cancer investigations such as fear of testing and diagnosis; perception of symptoms or the underlying urgency; and other demands in patients’ lives.^29^ Participants demonstrated some awareness of patient views (usually positive) of the RDC; however, recent research^30^ also highlighted areas of difficulty, which included limited patient understanding of the reasons for their referrals, that they were on a cancer pathway, and of the results of investigations.

### Implications for research and practice

The findings demonstrate that GPs value the pathway, which was felt to benefit both GPs and patients. The finding that not all GPs were aware of the pathway, despite extensive attempts to raise awareness, highlights the importance of ongoing and long-term publicity to maintain awareness of the pathway and any changes.

To date there is a limited, but emerging literature in respect of patients’ experiences with RDCs.^2,30,31^ Therefore, the inclusion of patients’ experiences, as reported by GPs, makes a valuable contribution to the current evidence base, while highlighting the need for further in-depth research with patients, to develop a more nuanced understanding of their experiences and any developments required. The perspectives of GPs and patients in areas of high deprivation require attention, as non-specific symptoms may be over-represented in such areas, and attendance barriers may be affected by sociodemographic factors.^26,29^

## Data

In the interests of participant anonymity, the authors cannot make anonymised transcripts available.
